# A Cross-Sectional Survey on HPV Vaccination in a Houston HIV Clinic

**DOI:** 10.3390/vaccines14040286

**Published:** 2026-03-24

**Authors:** Shailee R. Modi, Erika S. Fanous, Avery N. Sinnathamby, Laura O. Van Buskirk, Jason L. Holliday, J. Brooks Jackson, Mary B. Rysavy

**Affiliations:** 1 McGovern Medical School, University of Texas Health Science Center at Houston, Houston, TX 77030, USA; 2 Department of Obstetrics, Gynecology & Reproductive Sciences, University of Texas Health Science Center at Houston, Houston, TX 77030, USA; 3 Department of Pathology, University of Iowa, Iowa City, IA 52246, USA

**Keywords:** human papilloma virus, HPV vaccine, HIV

## Abstract

**Background/Objectives:** Human papillomavirus (HPV) is the most common sexually transmitted infection worldwide and causes cervical cancer in women. Patients with human immunodeficiency virus (HIV) are particularly susceptible to this virus. An effective vaccine against high-risk HPV genotypes is available. This study sought to evaluate barriers to HPV vaccination in HIV-positive female patients between the ages of 18 and 65 in a county clinic in Houston. **Methods:** A cross-sectional survey was conducted in May–June 2025 with 131 patients at Thomas Street Health Center in Houston. The survey assessed patient demographics, attitudes toward and knowledge of HPV vaccination (at least one dose), as well as self-reported cervical dysplasia and HPV infection history. Clinical data on available cervical dysplasia history were also gathered from the electronic medical record. Descriptive statistics were compiled, and comparisons between vaccinated and unvaccinated participants were performed using one-way analysis of variance for continuous variables and chi-square tests for categorical variables in R. **Results:** 75% of patients had prior knowledge of the HPV vaccine, but only 33% reported receiving at least one dose. The most common reason for not receiving the vaccine was never having been offered the vaccine by a provider. Separately, almost 40% of unvaccinated individuals had never heard of the vaccine. Of note, only 8.6% of respondents reported fully understanding the implications of vaccination and still choosing to decline. In this cross-sectional study, there was no statistically significant association between vaccination status and either recent dysplasia history in the electronic record or reported dysplasia or HPV infection history. Among eligible unvaccinated participants, 41% received the HPV vaccine after completing the survey. **Conclusions:** Addressing gaps in HPV vaccine communication and supporting clinicians in delivering confident counseling may improve vaccination rates in this at-risk population.

## 1. Introduction

Human papillomavirus (HPV) is the most common sexually transmitted disease worldwide. In the United States, there are 13 million new HPV infections each year [1]. HPV is a double-stranded, non-enveloped DNA virus first identified in 1956. More than 200 strains of HPV have been identified, with 16 and 18 being the highest risk strains that cause about 70% of cervical cancers worldwide, as well as other anogenital and oropharyngeal cancers [2,3]. However, other oncogenic HPV types like 31, 33, 35, 52, and 58 also contribute to cervical cancer and may account for a greater proportion of disease in immunocompromised populations [4].

Patients with human immunodeficiency virus (HIV) are at increased risk for persistent HPV infection and higher incidence of high-grade squamous intraepithelial lesions (HSIL), as immunosuppression impairs clearance [5]. In addition to immunosuppression, recent evidence suggests that the HIV trans-activator of transcription (Tat) protein may directly contribute to HPV-mediated carcinogenesis. Tat expression can persist despite effective antiretroviral therapy and has been associated with molecular changes that increase the invasive potential of HPV-related cervical lesions [6]. Cervical cancer is the most common cancer detected in HIV-positive women and is considered an AIDS-defining illness [7]. The incidence of cervical cancer is 3 to 4 times higher among women living with HIV compared with the general population [8].

The quadrivalent HPV vaccine, Gardasil^®^, was approved by the FDA in 2006 for ages 9–26 and protected against HPV types 6, 11, 16, and 18. In 2014, the nine-valent vaccine, Gardasil^®^ 9, was released, and covers the nine most common subtypes, with the potential to protect against 90% of cervical cancers [9]. In 2018, the FDA expanded approval of Gardasil^®^ 9 to include adults up to age 45, based on evidence of benefit in older age groups. The Advisory Committee on Immunization Practices (ACIP) issued a shared clinical decision-making recommendation for HPV vaccination in adults aged 27–45, emphasizing consideration of individual risk factors. Current CDC/ACIP guidance recognizes HIV-positive patients as high risk for HPV infection, and adults aged 27–45 may receive the vaccine after shared decision-making that considers potential risks and benefits [10]. Given that HIV-positive women are at elevated risk to develop cervical cancer after HPV infection, vaccination provides an important prevention opportunity in this population.

The HPV vaccine has been shown to be more than 95% effective in preventing HPV infection and cervical cancer lesions in patients who have never been infected by HPV strains included in the vaccine. For patients who have been previously exposed, Gardasil^®^ 9 still increases immune memory and offers protection against other HPV subtypes.

Notably, vaccine hesitancy has been on the rise with the advent of digital and social media platforms. HPV vaccination, in particular, has had lower uptake due to stigma around sexual activity and with the spread of misinformation regarding side effects [11,12]. Provider hesitancy may also contribute to lower vaccine uptake in adults. In a study of clinicians practicing in federally qualified health centers, 30% of respondents reported not discussing the HPV vaccine with patients aged 27–45 unless the patient initiated the conversation [13]. This practice reflects the shared decision-making model, in which providers may defer discussion to patients due to uncertainty about eligibility and potential benefit.

The Thomas Street Health Center in Houston was the first free-standing HIV clinic in the United States. The clinic provides comprehensive healthcare to predominantly low-income, HIV-positive patients as part of Harris Health, a county-run safety-net healthcare system. Despite the HPV vaccine being free and available on site, in 2023, only 7% of eligible patients had received any dose of the vaccine. The objective of this study was to evaluate barriers to HPV vaccination in HIV-positive patients and to identify areas of focus to achieve higher vaccination rates in this high-risk population going forward.

## 2. Materials and Methods

### 2.1. Study Population and Eligibility Criteria

A cross-sectional survey was conducted in May and June 2025 to assess attitudes, knowledge, and beliefs surrounding HPV vaccination among HIV-positive patients at the Thomas Street Health Center. Patients were eligible if they were cisgender women, transgender men, or were born with a uterus; were between the ages of 18 and 65; and spoke English or Spanish as their primary language. At the time of the study, any patient over the age of 52 would likely not have been eligible to receive the HPV vaccine, as the FDA approved the vaccine up to the age of 45 years in 2018. Despite this, patients 46–65 were included in the study to comprehensively evaluate knowledge, prior vaccine exposure, and attitudes towards vaccination in a population that may have been previously eligible or counseled under evolving guidelines.

A convenience sample was used to identify patients eligible for enrollment. Participants were approached before or after their appointments at various Thomas Street Health Center clinical specialties, including Gynecology, high-risk Obstetrics, Endocrinology, Rheumatology, Ophthalmology, Dermatology, and Infectious Disease/Primary Care. Informed consent was obtained for all participants. 

### 2.2. Survey Administration and Clinical Data Acquisition

The survey was administered using REDCap. Survey questions were developed de novo and included demographics (age, sex assigned at birth, marital status, sexual orientation, educational attainment), HPV vaccination knowledge (information sources, receipt of the vaccine), attitudes (intent to become vaccinated, reasons why patients received or declined vaccination), communication comfort (willingness to recommend the HPV vaccine to potential partners), and cervical dysplasia or HPV infection history (history of abnormal pap smear, additional procedures, previous positivity for HPV). Patients were classified as “vaccinated” if they confirmed previous receipt of the vaccine, regardless of the number of doses.

Patients were screened for eligibility based on age and sex-assigned-at-birth prior to approaching them for recruitment in their clinic rooms before or after their appointments. Patients were approached before or after their appointments in private clinic rooms. Attempts were made to approach each eligible patient, although some patients completed appointments before surveyors were able to approach them. Participation was voluntary and did not affect their clinical visit. Surveyors were uniformly trained to minimize interview bias. Questions were read aloud to participants with an interpreter if necessary, and participants stated their answers. A trained interpreter was used for patients speaking Spanish and American Sign Language. Survey length was approximately 5–7 min for English speakers and 8–10 min for Spanish speakers. Counseling about HPV and vaccination occurred after surveys were completed. Following survey administration and counseling about the vaccine, including safety, side effects, and prevention of cervical cancer and other HPV-related diseases, patients were asked if they wanted to receive the vaccine. If patients wanted to receive the vaccine, surveyors collaborated with their physician and nursing staff during their visit based on their eligibility criteria. Counseling provided by the surveyors did not replace that discussed by the medical care team, as it is standard in a clinical context.

Clinical data were obtained from the Epic electronic medical record regarding documented HPV vaccination, patients’ most recent pap smear history, HPV testing, and HIV viral load. Data were also collected on whether eligible patients received HPV vaccination at the clinic after participating in the survey.

### 2.3. Statistical Analysis

Statistical analysis was performed using R statistical software (version 2024.04.2 + 764). Descriptive statistics were generated to summarize demographic and clinical characteristics for the entire sample and then stratified by HPV vaccination status. The mean with standard deviation or the median with interquartile range was used for summarizing continuous variables. Comparisons between vaccinated and unvaccinated participants were performed using one-way analysis of variance (ANOVA) for continuous variables and chi-square tests for categorical variables. A *p*-value of less than 0.05 was considered statistically significant. A sensitivity analysis was performed to exclude participants who were unsure about prior vaccination status using the same statistical methods to assess for robustness of findings. Given the nature of this exploratory study, multivariable analysis was not performed to adjust for potential confounders, including age, education, and duration of HIV infection.

This study was approved by the Institutional Review Board at the University of Texas Health Science Center at Houston (HSC-MS-24-1004) prior to data collection. The Harris Health System Research & Sponsored Programs also granted administrative approval of the protocol. Data was stored securely and accessible only to the research team. 

## 3. Results

A total of 131 HIV-positive patients receiving care at Thomas Street Health Center completed the survey. The median age of participants was 46.7 (IQR 20–65) (Table 1). 61.8% self-identified as non-Hispanic African, Black, or African American, 30.5% self-identified as Hispanic, and 6.9% self-identified as White. Approximately 63% had been living with HIV for more than 10 years. Of those surveyed, a majority had a high school education or less, with 40% reporting a high school diploma as their highest level of education and 27.5% with less than a high school education. Among self-identified women, 90% identified as heterosexual and 3.8% as bisexual. Most participants were virally suppressed (<200 copies/mm^3^), although this was not associated with vaccination status. Results were similar in sensitivity analyses excluding participants who were unsure about prior vaccination status (“Unsure”).

**Figure 1:**
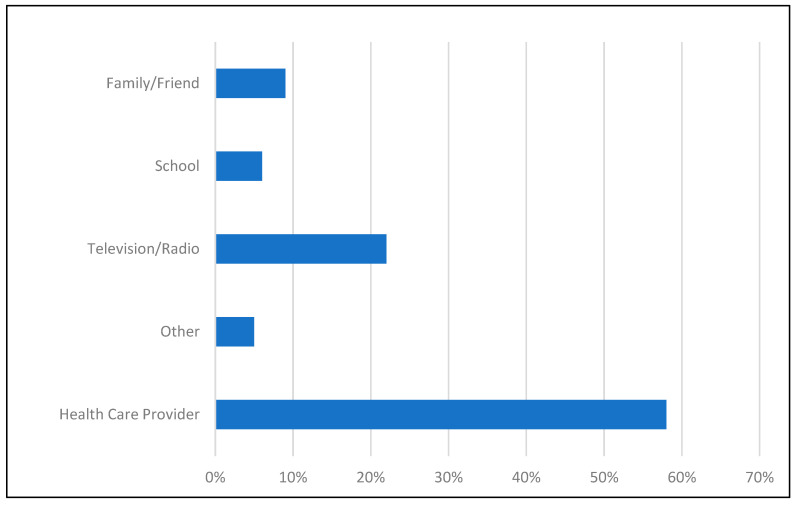
Sources of Information for Prior Knowledge of HPV Vaccine.

Overall, 75.6% had knowledge of the vaccine (Table 1), with 32.6% self-reporting at least one dose of the vaccine. Awareness of the vaccine was strongly associated with vaccination status. Participants who had heard of the HPV vaccine had significantly higher odds of being vaccinated compared to those who indicated they had not heard of the HPV vaccine (OR 11.05, 95% CI: 2.50–48.84, *p* < 0.001). Awareness of the HPV vaccine was higher among vaccinated individuals compared with unvaccinated (95.5% vs. 61.3%, *p* < 0.001). Among those aware of the vaccine, 58% had heard of it through their healthcare provider, followed by television/radio (22%), and family or friends (9%) (Figure 1). A few participants noted hearing about the vaccine through their children’s or grandchildren’s medical appointments.

**Figure 2:**
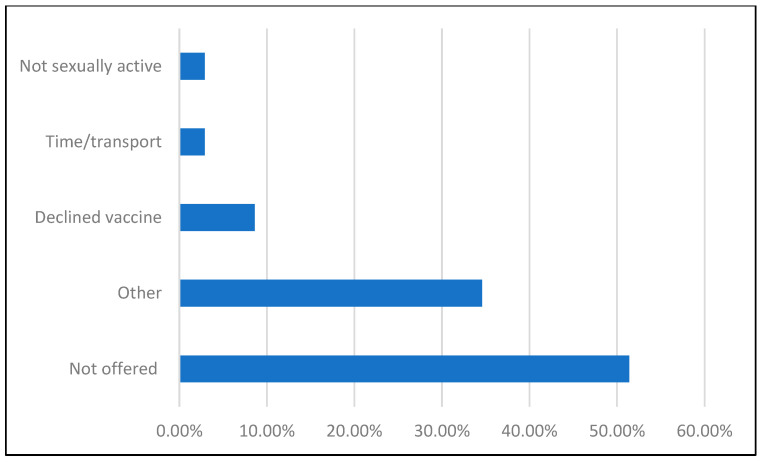
Reasons Patients Did not Receive the HPV Vaccine Prior to Survey.

The most common reason for not receiving the vaccination was that providers had not offered it (Figure 2). A small minority (8.6%) stated that they did not want the vaccine, and few participants (2.9%) expressed concerns about the time or transportation costs associated with multiple visits for HPV vaccination. Qualitative responses elaborated from the “Other” category discussed vaccine hesitation, lack of knowledge regarding side effects, and uncertainty about eligibility. Among eligible unvaccinated participants, 14.5% received HPV vaccination after completing the survey.

HPV vaccination of at least one dose was significantly associated with younger age (38.4 vs. 51.0 years, *p* < 0.001), though this should be interpreted as a proxy for eligibility. Open-ended responses describing reasons for vaccination commonly included a strong provider recommendation and a desire to prevent cancer. Of the 32 eligible and self-reported unvaccinated participants, 13 (40.6%) chose to receive the vaccination following survey administration (Table 1).

There was not a statistically significant association between vaccination status and a history of abnormal Pap smears or HPV-positivity (Table 2).

## 4. Discussion

Lack of knowledge regarding HPV vaccination was a significant barrier to HPV vaccination in this population of HIV-positive patients. There was higher awareness of the vaccine among those who were already vaccinated. The leading sources of information about HPV vaccination were healthcare providers, followed by TV/radio and family or friends.

Among unvaccinated respondents, the most cited reason for non-vaccination was never having been offered the vaccine. As a comparison point, 58% of respondents who were aware of the vaccine reported learning about it from a healthcare provider. These findings suggest that providers serve as key gatekeepers to HPV vaccination. We had expected a higher percentage of individuals to cite personal/cultural reasons for not receiving the vaccine and were surprised to find that most barriers could be addressed through education and communication. Additionally, 34.3% of participants selected “other” as their reason for not vaccinating; free-text responses after the other selection largely described misconceptions about how the vaccine works, further underscoring the role of education. Of note, only a small portion (8.6%) of respondents reported fully understanding the implications of vaccination but still choosing to decline. Following the survey, 41% of eligible patients chose to receive the HPV vaccine, underscoring the potential impact of patient education and provider engagement on vaccine uptake.

In the context of existing literature, these findings align with previous research emphasizing the role of the provider in vaccination decisions, including two other studies done by members of our research team [14,15]. A 2011 study by Rosenthal et al. found that the strength of a provider’s recommendation significantly influenced HPV vaccination uptake in women between 19 and 26 years of age; a strong provider recommendation resulted in a 4-fold increase in the likelihood of vaccination [16]. Provider recommendations may depend on clinician knowledge and comfort with vaccination counseling and shared clinical decision-making (SCDM). In a 2019 survey of U.S. primary care physicians, more than half reported uncertainty about key points to emphasize when discussing HPV vaccination with adults aged 27–45 years [17]. Physicians’ comfort with obtaining a sexual history may also affect counseling quality, as the physicians may not realize their patients have high-risk behaviors. Nearly half of physicians report not taking a sexual history during annual examinations [18]. Similarly, a 2019 study by Leung et al. found that some physicians lack confidence in addressing patients’ concerns about HPV vaccination, underscoring the need for targeted provider education. Potential interventions include designating a vaccine champion within clinics, implementing clinician prompts, and providing structured communication training to improve confidence in SCDM discussions [18,19].

In a 2022 study by Chambers et al. on HPV vaccination barriers in HIV-positive individuals in Ontario, Canada, vaccinated women (≥1 dose) were more likely to report familiarity with HPV compared with unvaccinated women (85.9% vs. 47.8%; *p* < 0.001) [20]. Similarly, in our study, vaccinated women demonstrated greater familiarity with the HPV vaccine than unvaccinated women (95.5% vs. 61.3%; *p* < 0.001). Chambers et al. also identified significant associations with employment, income, marital status, and immigration status. In contrast, we did not observe significant associations with transportation or socioeconomic barriers as primary factors driving lower vaccination rates, despite our study population coming from a socioeconomically challenged population in the county healthcare system. One potential explanation for this difference may be that HPV vaccination is not publicly funded for people living with HIV in Ontario, which could influence accessibility [21]. Conversely, at Thomas Street Health Center, the cost of vaccination is covered as part of routine care, so patients who attend the clinic are able to receive the vaccine even if transportation may at times pose a challenge in general. These findings suggest that while knowledge remains a key driver of vaccination, structural and financial barriers may vary by setting.

No statistically significant association was detected between vaccination status and either recent dysplasia history in the electronic record or reported dysplasia or HPV infection history. The lack of association with self-reported history may reflect recall bias. These findings should be interpreted cautiously, given the study’s limited statistical power and cross-sectional design. In the context of existing literature, recent population data show that unvaccinated women have a 2–3 times higher incidence of high-grade cervical lesions compared to vaccinated women [22]. Additionally, it is notable that our population had a high rate of abnormal pap smear. The finding that 48.9% of the population survey had a history of any abnormal pap smear highlights how high-risk this population is in general. For example, in one study in the general American population, only 20.3% of women had experienced an abnormal pap smear in their lifetime [23]. This again highlights our motivation for performing this study and working to improve vaccination for HPV in this population. Overall, our results indicate that the primary barrier to HPV vaccination is the lack of providers offering the vaccine, even in an HIV-positive county safety-net clinic population. Counseling on efficacy and side effects supports informed decision-making and reduces cervical cancer risk. Many patients reported not being offered the vaccine, while others cited misconceptions, such as believing the vaccine is unnecessary after testing positive for one HPV strain or thinking it is only for sexually active individuals. This emphasizes the important role providers continue to play in bridging knowledge deficits. The CDC recommends shared clinical decision-making for patients aged 26–45. Our data indicate these discussions may be infrequent, highlighting the need to support clinicians in facilitating HPV vaccination conversations with individuals living with HIV. Targeted support for clinicians to effectively engage HIV-positive patients in HPV vaccination discussions represents a key opportunity to improve preventative care.

Limitations of this study include selection bias, recall bias, interviewer bias, and the age distribution of participants, with a proportion over age 45 (53%) and therefore ineligible for vaccination. Additionally, this study’s findings apply to women living with HIV and may not be generalizable to male populations. Female participants were recruited during their clinical visit and participation was voluntary. Because of this, the sample may overrepresent patients more willing to engage in research or with more positive preexisting attitudes towards HPV vaccination. Vaccination status was categorized based on receipt of at least one dose, which does not distinguish between partial and series completion. Although the study included patients aged 18 to 65, individuals over the age of 52 at baseline were never eligible for the vaccine, as eligibility was limited to those under 26 between 2006 and 2018, and expanded to age 45 thereafter. Thus, the 33% vaccination rate of the surveyed population must be interpreted in the context of age-related eligibility. Another limitation is that data were collected from a single site, the Thomas Street Health Center, which has a large population of older adults. This likely reflects the clinic’s patient demographics, as many older individuals with HIV have multiple comorbidities and attend frequent appointments with various specialties located within the same facility.

Future public health implementation efforts should include expanding HPV vaccine counseling during postpartum visits. Pregnant individuals are often established with a clinic and demonstrate consistent adherence to appointments. The postpartum period represents a natural point for providers to reach unvaccinated patients within the eligible age range. Pregnancy and the postpartum period are also times in which many patients are attuned to making healthy life choices and may be more open to vaccination [24].

Future research directions should also include men living with HIV. In the United States, HPV vaccination is recommended for both men and women, though historically, and still in many other countries, males were/are not prioritized under the assumption that herd immunity could be achieved through widespread vaccination in women. However, this approach overlooks the increased risk of anogenital and/or oropharyngeal cancers in men who have sex with men. Men are less likely to receive information about the vaccine due to limited provider recommendations, misconceptions that the vaccine is only for females, and a general lack of awareness about HPV’s risks to men, including HPV-related cancers [25]. This is especially important for HIV-positive men as they are at higher risk of contracting HPV and having a harder time clearing the virus due to their immunocompromised state.

Future studies could also examine education level and language barriers and their effects on vaccination. Despite the use of certified interpreters, there were instances when patients misunderstood terms such as HIV versus HPV or expressed confusion due to limited formal education, with some reporting they had not completed schooling beyond the second grade. These areas represent important opportunities for improvement in education and inclusion in future interventions, such as visual aids or low-literacy infographics

## 5. Conclusions

The greatest barrier to HPV vaccination among HIV-positive patients at the Thomas Street Health Center in Houston was inadequate education about HPV and a lack of a provider recommendation. Our study demonstrated increased vaccine uptake following the survey. These findings suggest a clear opportunity to improve HPV vaccination rates through targeted provider education and improved patient counseling around the vaccine, thus potentially reducing preventable HPV-related illness in the HIV-positive population.

## Figures and Tables

**Table 1:** Demographic Information by Vaccination Status.

	Prior Vaccination (N = 44)	Unvaccinated (N = 75)	Unsure (N = 12)	Total (N = 131)	*p*-Value
**Age**					
Mean (SD)	38.4 (8.33)	51.0 (10.8)	50.8 (11.5)	46.7 (11.6)	<0.001
Median [Min, Max]	39.0 [20.0, 60.0]	53.0 [23.0, 65.0]	48.5 [32.0, 65.0]	47.0 [20.0, 65.0]	
**Age (Stratified)**					
≤45 years	37 (84.1%)	21 (28.0%)	3 (25.0%)	61 (46.6%)	<0.001
46–65 years	7 (15.9%	54 (72.0%	9 (75.0%)	70 (53.4%)	
**Race**					
African/Black/African American	28 (63.6%)	46 (61.3%)	7 (58.3%)	81 (61.8%)	0.494
Hispanic/Latino	13 (29.5%)	25 (33.3%)	2 (16.7%)	40 (30.5%)	
White	3 (6.8%)	3 (4.0%)	3 (25.0%)	9 (6.9%)	
Other	0 (0%)	1 (1.3%)	0 (0%)	1 (0.8%)	
**Highest Level of Education**					
Less than high school	9 (20.5%)	25 (33.3%)	2 (16.7%)	36 (27.5%)	0.581
High school diploma or GED	20 (45.5%)	28 (37.3%)	4 (33.3%)	52 (39.7%)	
Some college	6 (13.6%)	13 (17.3%)	3 (25.0%)	22 (16.8%)	
College graduate	8 (18.2%)	3 (4.0%)	2 (16.7%)	13 (9.9%)	
Post-Graduate	1 (2.3%)	1 (1.3%)	0 (0%)	2 (1.5%)	
Trade school	0 (0%)	5 (6.7%)	1 (8.3%)	6 (4.6%)	
**Length of HIV Diagnosis**					
<1 year	2 (4.5%)	7 (9.3%)	0 (0%)	9 (6.9%)	0.361
1–5 years	14 (31.8%)	11 (14.7%)	2 (16.7%)	27 (20.6%)	
5–10 years	2 (4.5%)	9 (12.0%)	0 (0%)	11 (8.4%)	
>10 years	25 (56.8%)	47 (62.7%)	10 (83.3%)	82 (62.6%)	
Missing	1 (2.3%)	1 (1.3%)	0 (0%)	2 (1.5%)	
**Heard of HPV vaccine**					
Yes	42 (95.5%)	46 (61.3%)	11 (91.7%)	99 (75.6%)	<0.001
No	2 (4.5%)	29 (38.7%)	1 (8.3%)	32 (24.4%)	
**Primary Language**					
English	31 (70.5%)	55 (73.3%)	9 (75%)	95 (72.5%)	0.916
Spanish	12 (27.3%)	20 (26.7%)	3 (25%)	35 (26.7%)	
Other	1 (2.3%)	0 (0%)	0 (0%)	1 (0.8%)	
**Received Vaccination Post-Survey**					
No	35 (79.5%)	19 (25.3%)	2 (16.7%)	56 (42.7%)	<0.001
Not eligible: >45 years	1 (2.3%)	42 (56.0%)	8 (66.7%)	51 (38.9%)	
Not eligible: pregnant	3 (6.8%)	1 (1.3%)	1 (8.3%)	5 (3.8%)	
Yes	5 (11.4%)	13 (17.3%)	1 (8.3%)	19 (14.5%)	
**Viral Suppressed (<200 copies/mm^3^)**					
Yes	7 (15.9%)	12 (16.0%)	0 (0%)	19 (45%)	0.519
No	36 (81.8%)	63 (84.0%)	12 (100%)	111 (84.7%)	

**Table 2:** Cervical dysplasia Screening results by vaccination status.

	Prior Vaccination (N = 44)	Unvaccinated (N = 75)	Unsure (N = 12)	Total (N = 131)	*p*-Value
**Most recent pap-smear in medical record**					
Abnormal	19 (43.2%)	21 (28.0%)	5 (41.7%)	45 (34.4%)	0.574
Normal	22 (50.0%)	43 (57.3%)	7 (58.3%)	72 (55.0%)	
Missing	3 (6.8%)	11 (14.7%)	0 (0%)	14 (10.7%)	
**Any abnormal Pap Smear (reported)**					
Yes	21 (47.7%)	34 (45.3%)	9 (75.0%)	64 (48.9%)	0.558
No	21 (47.7%)	38 (50.7%)	2 (16.7%)	61 (46.6%)	
Unsure	2 (4.5%)	3 (4.0%)	1 (8.3%)	6 (4.6%)	
**Any history of HPV Positivity (reported)**					
Yes	5 (11.4%)	12 (16.0%)	2 (16.7%)	19 (14.5%)	0.927
No	35 (79.5%)	57 (76.0%)	8 (66.7%)	100 (76.3%)	
Unsure	4 (9.1%)	5 (6.7%)	2 (16.7%)	11 (8.4%)	
Missing	0 (0%)	1 (1.3%)	0 (0%)	1 (0.8%)	

## Data Availability

Original data is available upon request.

## Abbreviations

HPV	Human Papillomavirus
HIV	Human Immunodeficiency Virus
